# High-Performance Solid-State Supercapacitors Fabricated by Pencil Drawing and Polypyrrole Depositing on Paper Substrate

**DOI:** 10.1007/s40820-015-0039-3

**Published:** 2015-04-10

**Authors:** Jiayou Tao, Wenzhen Ma, Nishuang Liu, Xiaoliang Ren, Yuling Shi, Jun Su, Yihua Gao

**Affiliations:** 1 Wuhan National Laboratory for Optoelectronics (WNLO) & School of Physics, Center for Nanoscale Characterization & Devices (CNCD), Huazhong University of Science and Technology, Wuhan, 430074 People’s Republic of China; 2 School of Physics and Electronics, Hunan Institute of Science and Technology, Yueyang, 414006 People’s Republic of China

**Keywords:** Supercapacitor, Paper, Pencil drawing, Polypyrrole

## Abstract

A solid-state powerful supercapacitor (SC) is fabricated with a substrate of Xerox paper. Its current collector based on a foldable electronic circuit is developed by simply pencil drawing. Thin graphite sheets on paper provide effective channels for electron transmission with a low resistance of 95 Ω sq^−1^. The conductive organic material of polypyrrole coated on thin graphite sheets acts as the electrode material of the device. The as-fabricated SC exhibits a high specific capacitance of 52.9 F cm^−3^ at a scan rate of 1 mV s^−1^. An energy storage unit fabricated by three full-charged series SCs can drive a commercial light-emitting diode robustly. This work demonstrated a simple, versatile and cost-effective method for paper-based devices.

**Electronic supplementary material:**

The online version of this article (doi:10.1007/s40820-015-0039-3) contains supplementary material, which is available to authorized users.

## Introduction

Supercapacitors (SCs), as the promising energy storage devices, have attracted tremendous attention for a set of features, such as high power density, fast rates of charge–discharge process, long cycling life and improved safety [1–3]. Particularly, SCs can provide much higher power density than batteries and higher energy density than conventional capacitors, which bridge the gap between those two kinds of typical energy storage devices [4–6]. According to the underlying energy storage mechanism, SCs can be classified into two categories [4, 5]. One is electrochemical double-layer capacitors (EDLCs) which store electrical energy by electrostatic accumulation of charges between the surfaces of the electrode materials and electrolyte [4]. Although EDLCs exhibit ultrahigh power density and distinguished long-term cycling performance, the stored energy is limited by the finite electrical charge separation at the interface between electrolyte and electrode materials [3]. The other type of SC is the so-called pseudocapacitor, which stores energy due to fast and reversible redox reactions occurring on the surface or near surface of the active electrode materials. Compared to EDLCs, pseudocapacitors have high energy density but low power density and short cycle life [6].

Paper is inexpensive, foldable, environmentally benign nature and widely used in our daily life. Commonly, paper is composed of cellulose fibres with a typical diameter of about 20 μm. In recent years, paper is becoming a promising flexible substrate for various electronics, such as solar cells [7], transistors [8], displays [9] and energy storage devices [10–12]. The realization of paper-based devices is highly desired not only for their wide range of applications, but also for their compatibility with printed electronics. Aimed at low cost, environmentally benign nature and wide range of applications, paper was exploited for the substrate of our SCs. Carbon materials, as the most typical electrode materials for EDLCs, have been extensively studied in the past decades due to their good conductivity, robust mechanical character and stable electrochemical behaviour [13–17]. Among carbon materials, graphene has some fascinating features, such as large surface area, high flexibility, excellent conductivity and good chemical/thermal stability [18, 19]. However, high temperature and vacuum are needed during the synthesis process of graphene [20–23]. Consequently, some unfavourable issues emerge such as high cost, elaborate fabrication or difficulty in large-scale fabrication. Herein, we got inspiration from ordinary writing manners and successfully drew arbitrary shapes of current collectors for our SCs using a pencil. Multilayered graphene (thin graphite sheets) was transferred onto the paper substrates during this simple process, which provided an effective transmission path for electrons. For the sake of enhancing the electrochemical performance of the devices, polypyrrole (PPy) was deposited on the pencil drawing paper, which was also used as the pseudocapacitive material in this research. Compared to other conductive polymers, PPy has greater density and a great degree of flexibility in electrochemical process [24, 25], which result in a high volumetric capacitance and high mechanical performance. After the deposition of PPy, two PPy thin graphite sheet paper electrodes were assembled with a gel electrolyte of H_3_PO_4_/polyvinyl alcohol (PVA). The as-fabricated solid-state SCs exhibited good flexibility and a high specific capacitance of 52.9 F cm^−3^ at a scan rate of 1 mV s^−1^, which is much higher of some SCs than in prior literatures [26, 27]. This technique represents a low cost, applied and versatile fabrication method for paper-based energy devices.

## Experiment

To get paper-based SCs, a piece of Xerox paper (1.5 cm^2^) was drawing by a 4B pencil (86 % graphite and 14 % clay) until its sheet resistance reduced to about 95 Ω sq^−1^ (~150 times of scratching). After that, a layer of thin graphite sheets was deposited on the paper. Then, the graphite–paper composite (the area is about 1.0 × 1.0 cm) was immersed in a solution that contained 0.2 M NaClO_4_ and 5 % (V:V) pyrrole monomer, and PPy was grown on the drawn paper via an electrochemical deposition process. Three-electrode configuration was used in this deposition process with Ag/AgCl as the reference electrode, platinum foil as the counter electrode and the drawn paper as the working electrode. A constant voltage of 0.8 V was applied during the process. Then the as-grown sample was washed with deionized water and dried at room temperature. In order to seek for the dependence of SC performance on PPy deposition time, the deposition time of PPy on the drawn paper was different. Finally, two pieces of 1.0 × 1.5 cm functionalized paper were used as electrodes with the opposite area of 1.0 × 1.0 cm. A gel composite H_3_PO_4_/PVA was used which acted as the separator and the electrolyte between the two electrodes. After the gel electrolyte dried completely, the quasi-solid-state SC was prepared.

## Results and Discussions

**Fig. 1:**
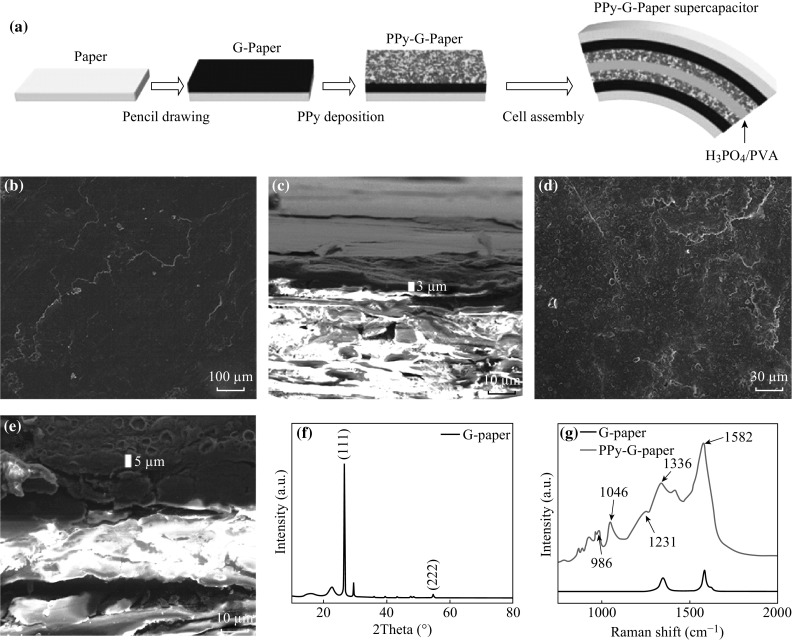
**a** Schematic of fabrication process of the PPy-G-paper. **b** A SEM image of the G-paper. **c** A cross-sectional SEM image of the G-paper. **d** A SEM image of PPy-G-paper. **e** A cross-sectional SEM image of the PPy-G-paper. **f** XRD of the G-paper illustrating the graphite. **g** Raman spectra of the G-paper and PPy-G-paper

The fabrication process is illustrated in Fig. 1a. Scanning electron microscopy (SEM) images of the electrode show that the drawing on paper with 4B pencil produced multilayer graphene coating on the substrate (Fig. 1b). The cross-section SEM image shows that the thickness of the graphite film is about 3.0 µm (Fig. 1c), and the graphite has been coated on the paper surface tightly. Figure 1d shows that a layer of PPy has been polymerized and wrapped on the drawing paper. There are a certain amount of micro- or nanopores on the surface, which provides larger effective area for redox reaction during charge–discharge section (Fig. S1). The thickness of the active materials (graphite and PPy) also has been measured to be about 5.0 µm (after 5-min electrodeposition of PPy) and they tightly attach to each other (Fig. 1e). X-ray diffraction (XRD) of graphite paper (G-paper) was also carried out (Fig. 1f). The peaks at 26.67° and 54.83° fit well with graphite (111) and (222), respectively. It further confirmed that the film made by pencil drawing mainly contained graphite. To study the functional groups information, Raman spectra of both G-paper and PPy-G-paper are shown in Fig. 1g. The Raman spectrum of G-paper exhibits two prominent peaks. In detail, the peak at 1380 cm^−1^ is designated as the well-documented D band owing to the disorder-induced mode from Raman scattering at the graphene edges [28], and the peak at 1618 cm^−1^ is attributed to the doubly degenerate in-plane *E*_2g_ vibration mode. In the Raman spectrum of the PPy-G-paper, three typical peaks arising from PPy can be indexed. The peak at 1582 cm^−1^ is assigned to C=C back-bone stretching attributed to the G band of graphene. The peak at 1336 cm^−1^ corresponds to the D band of graphene. The peak at 986 cm^−1^ is assigned to ring vibration of PPy [29], whereas bands at 1046 and 1231 cm^−1^ are due to C–H stretching. The Raman spectra confirmed the presence of PPy and graphite in the composite film, forming a PPy-G-paper hybrid structure.

**Fig. 2:**
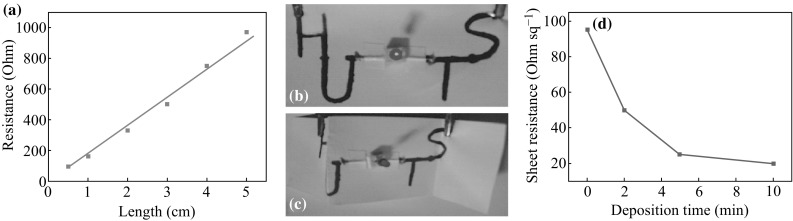
**a** The resistance measurement of the G-paper (width of the stripe is 0.5 cm) at different lengths. **b** The application of driving an LED with the G-paper circuit. **c** To drive an LED by the G-paper circuit under negative and positive angle folding. **d** The sheet resistance of PPy-G-paper at different PPy deposition times

The electric resistance of G-paper was measured at different lengths (all the width of the measured G-paper is 0.5 cm). It reveals that there is a linear correlation between the length and the distance (Fig. 2a). The results indicated that thin graphitic sheets coated on paper by pencil drawing have good conductivity, which provide a stable transmission channel for electrons during charge–discharge process. For convenient portability, some paper-based applications require electronic circuits that could be folded irrespective of whether the folding angle is negative or positive. We have fabricated a simple foldable circuit which could drive a light-emitting diode (LED) under negative or positive folding angles (Fig. 2b, c). The small change of circuit resistance allows the paper-based circuit board to be folded at any angle. It illustrates that the graphite on paper provides a good conductivity. After the PPy deposition, the sheet resistance substantially reduced from 95 to 21.3 Ω sq^−1^ with the PPy deposition time increased from 0 to 10 min (Fig. 2d). Obviously, the sheet resistance decreased slightly when the deposition time increased from 5 to 10 min. This good conductivity also can be confirmed in the following Nyquist plot.

**Fig. 3:**
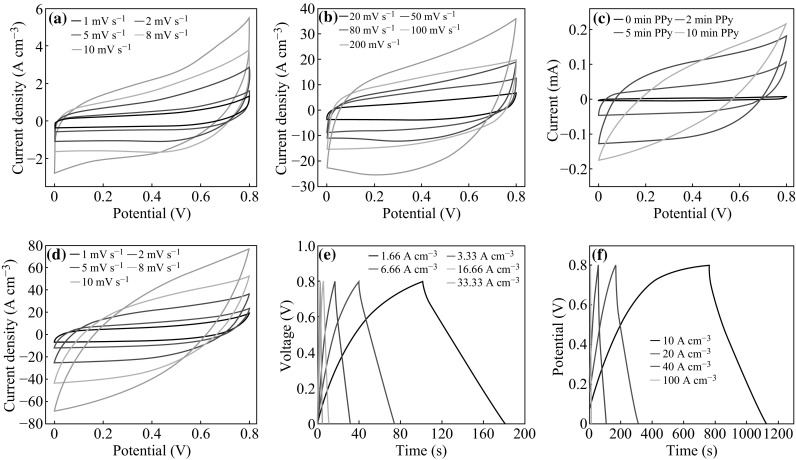
CV curves of **a** G-paper SC with the scan rate from 1 to 10 mV s^−1^ and **b** from 20 to 200 mV s^−1^, **c** The devices with different PPy deposition times at a scan rate of 5 mV s^−1^, **d** The PPy-G-paper SC with the scan rate from 1 to 10 mV s^−1^. GCD behaviour of **e** a G-paper-based SC and **f** a PPy-G-paper-based SC at different current densities

To explore the electrochemical performance of the PPy-G-paper SCs, a typical two-electrode configuration has been employed in this work. All electrochemical measurements were carried out at room temperature. We performed the cyclic voltammogram (CV) scans of the G-paper electrodes at different scan rates from 1 to 200 mV s^−1^ (Fig. 3a, b). The CV curve kept in a near rectangular shape even at the scan rate of 100 mV s^−1^ indicates that the device has good capacitive performance. The CV curves during higher power process are shown in Fig. S2. The CV of PPy-G-paper at different PPy deposition times was also measured (as shown in Fig. 3c and Fig. S3). When the deposition time increased to 5 min, the capacitances of the PPy-G-paper SCs also had increased and there was a downward trend after 5-min PPy deposition. It indicates that the device with 5 min of PPy electrodeposition has better performance than that with 2 and 10 min. So the optimized deposition time of PPy is 5 min. Figure 3d clearly shows the excellent CV performance at different scan rates of the PPy-G-paper SCs with 5 min of PPy electrodeposition. The device has a high specific capacitance of 52.9 F cm^−3^ at 1 mV s^−1^, which is much higher than those reported in prior literatures [26, 27]. We supposed that the high specific capacitance value should be attributed to the synergetic effect of graphite and PPy conductive wrapping layer, which improved the electrical conductivity and acted as the pseudocapacitance materials simultaneously. To further evaluate the electrochemical performance of the SCs, the galvanostatic charge/discharge (GCD) characterization was performed with different current densities over the voltage window of 0–0.8 V (in Fig. 3e, f). The near linear voltage versus time profiles and the near symmetrical charge/discharge characteristics represent good capacitive characteristics of both G-paper SCs and PPy-G-paper SCs. Comparing Fig. 3e with Fig. 3f, it is clear to see that the capacitance of the PPy-G-paper SC is significantly improved than that of the G-paper SC.

**Fig. 4:**
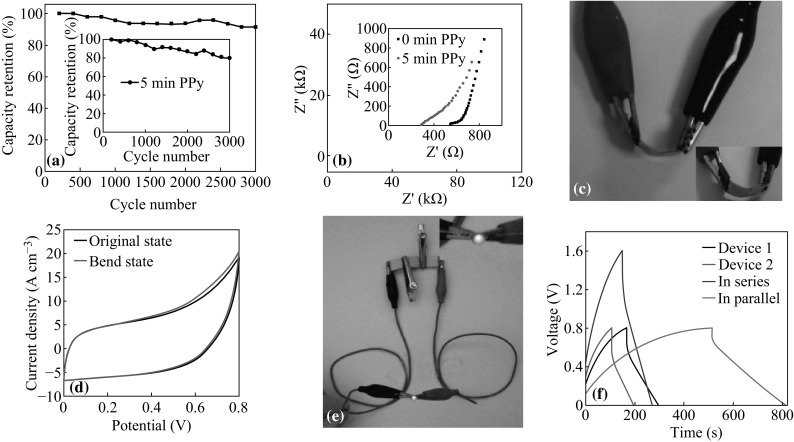
**a** Capacity retention ratios of a G-paper-based SC (the *inset* is of a PPy-G-paper-based SC). **b** The Nyquist plot of the G-paper- and PPy-G-paper-based SCs. **c** The photographic image of bending state. **d** CV curves of the PPy-G-paper-based SC at bending state. **e** Optical images of the three SCs in series driving an LED. **f** GCD curves of single SC and two SCs connected in series or in parallel

Cycling life is an important parameter to determine the performance of SCs. In order to study the electrochemical stability, the cycling performance of the as-fabricated SCs was tested. Figure 4a shows that the SC based on G-paper electrodes has excellent cycling performance with over 90 % retention of capacity after 3000 cycles. For the device based on PPy-G-paper, 80.5 % of the initial capacitance was retained after 3000 cycles (the inset of Fig. 4a). Figure 4b shows the Nyquist plots in the frequency range from 100 kHz to 0.01 Hz with the potential amplitude of 10 mV. The equivalent series resistance (ESR) of the G-paper and PPy-G-paper reduces from 536.6 to 280.8 Ω. It revealed that the conductivity was improved after PPy deposition.

In order to demonstrate the flexibility of our devices, the bent state is shown in Fig. 4c. We also test the CV performance at original/bent state; as shown in Fig. 4d, the CV curves of the device just have a little influence. An example for the application of the connected SCs is shown in Fig. 4e and the inset, where three arbitrary PPy-G-paper-based SCs are connected in series. They can drive a commercial LED (Fig. 4e) as an energy source when it has been fully charged. We picked two SCs (devices 1 and 2) and measured their capacitances, which are 16.1 and 11.3 mF at the current density of 20 A cm^−3^, respectively. As shown in Fig. 4f, when they are connected in series, the capacitance of the whole device is calculated to be 7.6 mF, when in parallel, it is 32.8 mF. The results reveal that the capacitance of the connected SCs roughly obeys the basic rule of series and parallel connections of capacitors. So we can take various connections of our SCs to meet a wide variety of demands in practice.

## Conclusions

In summary, we fabricated SCs on Xerox paper using pencil drawing and PPy deposition successfully. The thin graphite sheets drawn by pencil acted as a good EDLC material and a good current collector. The SCs based on PPy-G-paper electrodes showed high specific capacitance of 52.9 F cm^−3^ at a scan rate of 1 mV s^−1^. In addition, three SCs connected in series can drive a commercial LED. This method of fabricating the energy storage devices is of low cost and environment friendly, and the paper SCs can potentially guide the development of paper electronics for its low cost and high compatibility.

## Electronic supplementary material

Below is the link to the electronic supplementary material.

Supplementary material 1 (DOC 4344 kb)
