# Supplementary material for: High-Performance Solid-State Supercapacitors Fabricated by Pencil Drawing and Polypyrrole Depositing on Paper Substrate
Source: Nanomicro Lett. 2015 Apr 10;7(3):276–81. doi: 10.1007/s40820-015-0039-3 (PMC6223895; doi:10.1007/s40820-015-0039-3)
Supplement: Supplementary file 1 — Supplementary material 1 (DOC 4344 kb) [file 40820_2015_39_MOESM1_ESM.doc]

Supplementary Information for

High Performance Solid-state Supercapacitors Fabricated by Pencil Drawing and Polypyrrole Depositing on Paper Substrate

Jiayou Tao^1,2,†^, Wenzhen Ma^1,†^, Nishuang Liu^1^, Xiaoliang Ren^1^, Yuling Shi^1^, Jun Su^1^, Yihua Gao^1^,*

^1^Center for Nanoscale Characterization & Devices (CNCD), Wuhan National Laboratory for Optoelectronics (WNLO) & School of Physics, Huazhong University of Science and Technology, Wuhan 430074, People’s Republic of China

^2^School of Physics and Electronics, Hunan Institute of Science and Technology, Yueyang 414006, People’s Republic of China

^†^The authors contributed equally to this work.

*Corresponding author. E-mail: [gaoyihua@hust.edu.cn](mailto:gaoyihua@mail.hust.edu.cn)

Figure Captions

Fig. S1 SEM images of PPy surface

Fig. S2 CV curves of G-paper electrodes at a scan rate of 0.5, 0.8, and 1 v s^-1^, respectively

Fig. S3 CV curves of PPy-G-paper electrodes at a scan rate of 8 mv s^-1^


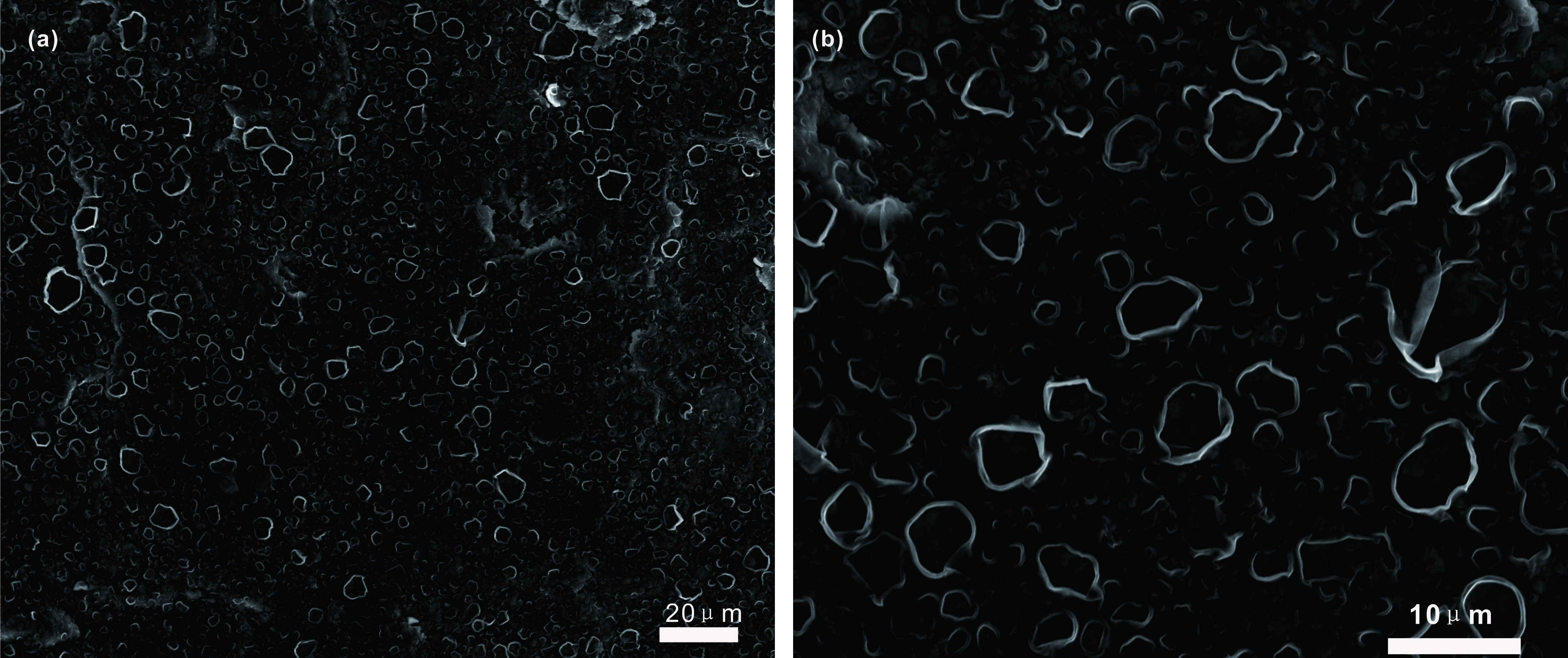


Fig. S1


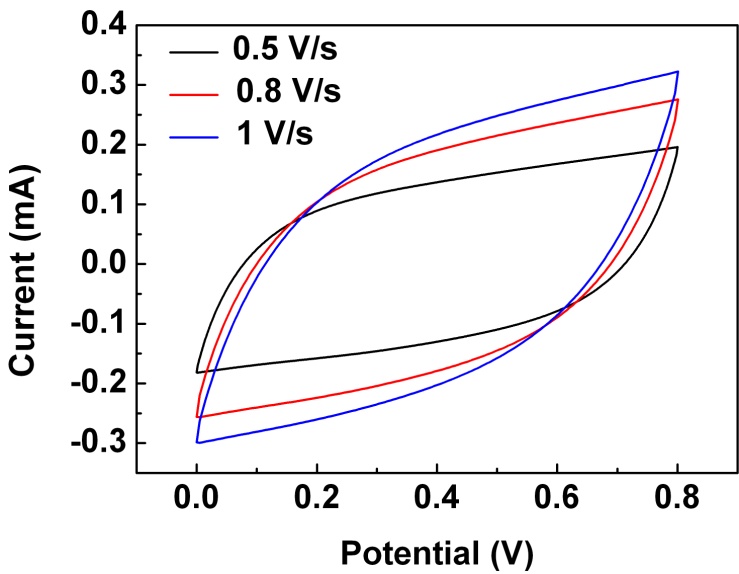


Fig. S2


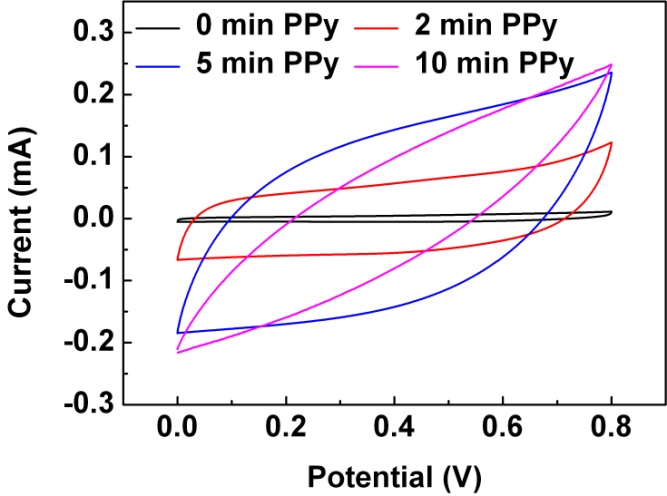


Fig. S3
